# Advances and Functional Integration of Hydrogel Composites as Drug Delivery Systems in Contemporary Dentistry

**DOI:** 10.3390/gels10100661

**Published:** 2024-10-16

**Authors:** Dragos Nicolae Fratila, Dragos Ioan Virvescu, Ionut Luchian, Monica Hancianu, Elena Raluca Baciu, Oana Butnaru, Dana Gabriela Budala

**Affiliations:** 1 Department of Oral Diagnosis, Faculty of Dental Medicine, “Grigore T. Popa” University of Medicine and Pharmacy, 700115 Iasi, Romania; 2 Department of Dental Materials, Faculty of Dental Medicine, “Grigore T. Popa” University of Medicine and Pharmacy, 700115 Iasi, Romania; 3 Department of Periodontology, Faculty of Dental Medicine, “Grigore T. Popa” University of Medicine and Pharmacy, 700115 Iasi, Romania; ionut.luchian@umfiasi.ro; 4 Department of Pharmacognosy, Faculty of Pharmacy, “Grigore T. Popa” University of Medicine and Pharmacy, 16 University Street, 700115 Iasi, Romania; 5 Department of Biophysics, Faculty of Dental Medicine, “Grigore T. Popa” University of Medicine and Pharmacy, 700115 Iasi, Romania; 6 Department of Prosthodontics, Faculty of Dental Medicine, “Grigore T. Popa” University of Medicine and Pharmacy, 700115 Iasi, Romania

**Keywords:** hydrogels, nanomaterials, biocompatibility, dental applications, periodontitis

## Abstract

This study explores the recent advances of and functional insights into hydrogel composites, materials that have gained significant attention for their versatile applications across various fields, including contemporary dentistry. Hydrogels, known for their high water content and biocompatibility, are inherently soft but often limited by mechanical fragility. Key areas of focus include the customization of hydrogel composites for biomedical applications, such as drug delivery systems, wound dressings, and tissue engineering scaffolds, where improved mechanical properties and bioactivity are critical. In dentistry, hydrogels are utilized for drug delivery systems targeting oral diseases, dental adhesives, and periodontal therapies due to their ability to adhere to the mucosa, provide localized treatment, and support tissue regeneration. Their unique properties, such as mucoadhesion, controlled drug release, and stimuli responsiveness, make them ideal candidates for treating oral conditions. This review highlights both experimental breakthroughs and theoretical insights into the structure–property relationships within hydrogel composites, aiming to guide future developments in the design and application of these multifunctional materials in dentistry. Ultimately, hydrogel composites represent a promising frontier for advancing materials science with far-reaching implications in healthcare, environmental technology, and beyond.

## 1. Introduction

Hydrogels play a crucial role in dentistry by offering targeted drug delivery, promoting tissue regeneration, and supporting wound healing. Their unique properties, such as biocompatibility, flexibility, and responsiveness to the oral environment, make them essential for addressing complex dental conditions like periodontitis, for tissue regeneration, and they enhance wound healing, thus improving patient care.

The oral cavity is the start of the digestive tract and is essential for both alimentation and communication, significantly impacting daily life [1]. Numerous disorders, including infections and cancers, can manifest in the oral cavity, adversely affecting human health and vitality [2]. Oral illnesses, including caries, pulpitis, periodontitis, periimplantitis, and oral malignancies, typically present as microbial infections, localized inflammation, and tissue degeneration [3,4]. Managing various oral disorders is crucial for maintaining oral health, general well-being, and quality of life [5].

Despite substantial advancements in contemporary dental medicine, the efficacy of existing therapies remains predominantly limited by the characteristics of the dentition and biomaterials employed [6]. Resin composites employed in dental cavity restorations may undergo microleakage over time, resulting in biofilm growth and the onset of secondary caries [7]. Moreover, root canal filling materials employed to treat pulp diseases can inhibit reinfection but do not enhance the fragility of devitalized teeth [8]. Conventional periodontal treatments, including scaling and root planning, may result in adverse consequences such as discomfort and localized dental damage [9]. Antibiotic dressings employed to treat periodontitis may induce side effects, including photosensitivity and lasting tooth discoloration [10]. Additionally, bone grafts employed to substitute damaged periodontal bone and remedy maxillofacial defects may induce difficulties at the donor’s site, including infection and additional injuries [11]. Administering anticancer medications carries the potential for systemic adverse effects [12]. Consequently, there is an increasing need for new biomaterials to tackle issues in oral and craniofacial treatment and regeneration [13].

**Figure 1:**
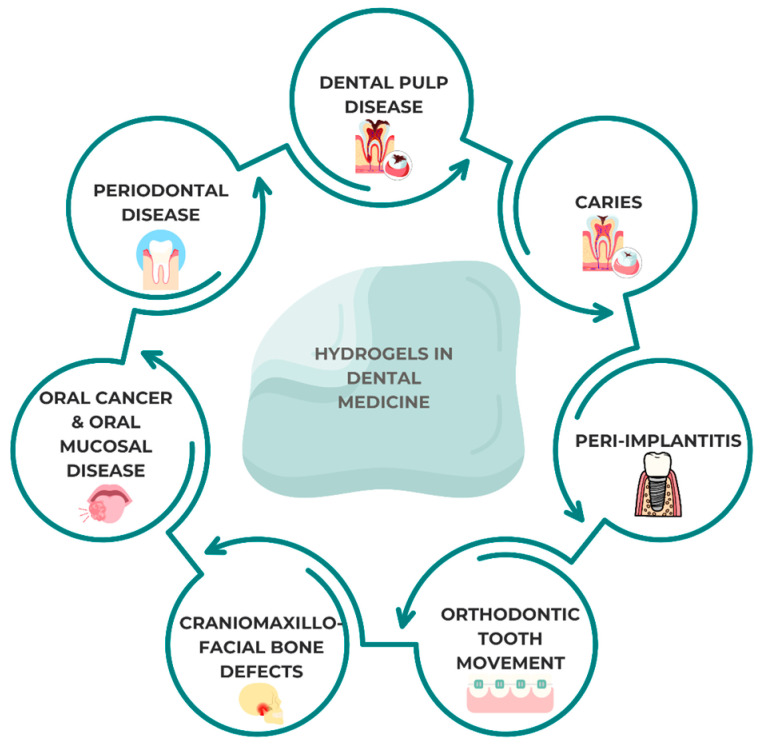
Potential uses of hydrogels in the dental field.

As hydrogels resemble the structure of natural extracellular matrices, they are considered as ideal scaffold materials in the tissue engineering sector for treating dental pulp and periodontal lesions. Combined with different kinds of stem cells and growth hormones, diverse hydrogel complexes have played a positive role in endodontic and periodontal tissue engineering investigations. Furthermore, hydrogels display stimuli-responsive behavior to external stimuli, resulting in hydrogels having a promising application in local medication administration; all of the above are presented in Figure 1. Stimuli-responsive behavior refer to the ability of hydrogels to react or change their physical or chemical properties in response to external stimuli, such as changes in pH, temperature, light, or enzyme activity. This responsiveness enables hydrogels to release medication in a controlled manner, making them particularly effective for local drug delivery applications.

There are distinct challenges in the advancement of materials for healthcare purposes. Innovations include shape-recoverable hydrogels for cosmetic applications and chitin hydrogel/nanocomposite scaffolds for periodontal health to enhance the limits of existing treatment efficacy and ease of application [14]. Moreover, developing materials that demonstrate both bioactivity and biomechanical compatibility with physiological tissues is essential for seamless integration and functionality within the human body [15].

## 2. Historical Development of Hydrogels

Hydrogels look like real tissue due to their high water content, porous structure, and supple texture. In 1960, Wichterle and Lim initially created hydrogels as biomaterials. They created a synthetic hydrogel using poly2-hydroxyethyl methacrylate (PHEMA) as a filler for eye enucleation and contact lenses [16]. From the 1970s to the 1990s, many preliminary studies [17,18,19,20,21] raised the cost of hydrogels for drug delivery and controlled release of active compounds, which is the process by which hydrogels release biologically active substances, such as drugs, proteins, peptides, or other therapeutic agents, in a controlled manner. These bioactive chemicals interact with the biological environment to produce a specific therapeutic effect, such as reducing inflammation, promoting tissue regeneration, or combating infections. Throughout the past six decades, hydrogels have been developed for various applications, including implantable, injectable, and sprayable forms suitable for numerous organs and tissues [19,20]. Hydrogels have recently attracted significant interest in the domains of environmental engineering, soft robotics, and wastewater treatment [22,23,24]. With the advancement in hydrogel technology, these materials are anticipated to be applied in a broader range of fields. Scientists continue to show significant interest in the biomedical applications of hydrogels, as demonstrated by the 25,000 references to hydrogels for biomedical applications in the past five years.

**Figure 2:**
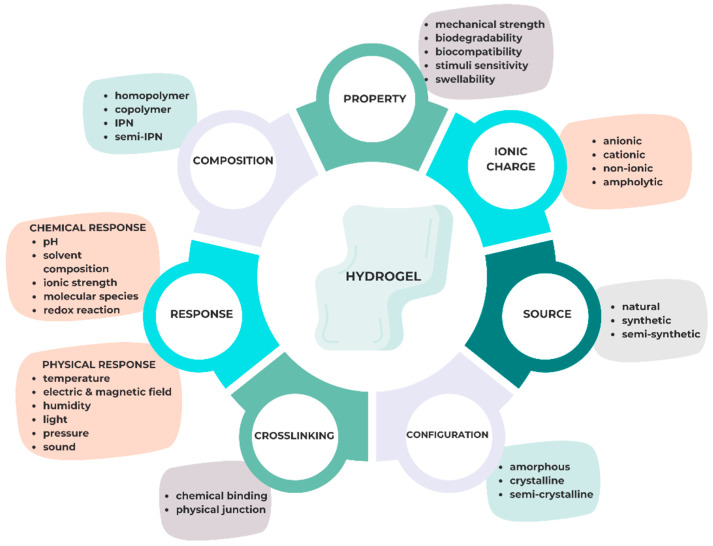
Hydrogel characteristics and classification (with IPN—interpenetrating polymer network).

Changing the hydrophilic to hydrophobic ratio, the concentration of the initiator or polymer, and the reaction conditions (time, temperature, container, etc.) can alter the properties of hydrogels, such as their swelling–deswelling rate, stiffness, degradability, and mech size [25,26,27,28]. The fundamental concepts are illustrated in Figure 2.

Drug delivery, tissue engineering, and regenerative medicine are just a few of the medical applications that could benefit greatly from these materials’ ability to mimic the extracellular matrix (ECM) [29,30]. The use of nanohydroxyapatite and clays in hydrogel composites improves their mechanical properties and functionality, making them more versatile [30,31]. Innovative hydrogels can now change their characteristics in response to changes in temperature and pH, for example, thanks to developments in hydrogel technology [31]. From injectable hydrogels for targeted therapy to superabsorbent hydrogel composites for advanced medical applications, hydrogels are adaptable and closely resemble biological tissues, making them a cornerstone in the development of innovative medical treatments and devices [32,33]. Biological tissues are the natural structures and materials that make up the organs and systems of living organisms, such as skin, muscles, cartilage, and mucous membranes; hydrogels closely resemble biological tissues because they share similar properties, such as a high water content, softness, elasticity, and mechanical behavior.

## 3. Hydrogels’ Composition and Properties

A hydrogel is a three-dimensional (3D) network of polymer materials that can reversibly absorb and release a considerable amount of water or other solvents without dissolving. There are hydrophilic functional groups (OH, CONH, COOH, CONH_2_, SO_3_H, and NH_2_) in the polymer chain that can attract solvents. The solvents can then migrate into the polymeric network, filling the space between macromolecules and causing the hydrogel to expand and occupy a larger volume (hydrogel swelling) [30,31].

The swelling process, release velocity, and kinetics of the absorbed fluid and active component are influenced by the chemical composition and density of the hydrogels’ network. Because they produce physical interactions both within and between molecules, such as hydrogen bonds, hydrophobic contacts, and dipole–dipole interactions, it is crucial for functional groups to have a lot of spare space [32].

In reaction to specific chemical and physical stimuli, hydrogels go through a dramatic change in volume, often known as a gel–sol phase transition. Chemical or biochemical stimuli include pH, ions, and certain chemical compositions; physical stimuli include temperature, electric and magnetic fields, solvent composition, light intensity, and pressure [33]. However, these conformational changes are usually reversible, so hydrogels can return to how they were before a reaction as soon as the trigger is taken away. Factors such as the type of monomer, charge density, pendant chains, and degree of cross-linkage largely dictate how hydrogels react to outside stimuli. The amount of an external stimulus has a direct correlation with the amplitude of the response [34,35].

Hydrogel composites have become a crucial component in medical and scientific research, particularly in regenerative medicine, tissue engineering, and drug delivery systems [34]. Although these materials possess significant potential, their application presents obstacles that require continuous innovation to realize their full capabilities [35].

Hydrogels are complex networks of crosslinked polymers with hydrophilic characteristics that have remarkable properties such as hydrophilicity, biocompatibility, porosity, and viscoelasticity due to their ability to absorb significant quantities of water while preserving insoluble components [36]. Covalently crosslinked networks are referred to as “chemical” or “permanent” gels. The crosslink density and polymer–water interaction parameter determine their equilibrium swelling state. When molecular entanglements and secondary forces like ionic, H-bonding, or hydrophobic interactions hold the networks together, the gel is referred to as a “physical” or “reversible” gel. Physical changes or stress can interrupt any of these interactions, and they are all reversible [36,37].

Hydrogels can transport medicines and cells and replicate the biochemistry of the extracellular matrix (ECM) [37,38]. For these reasons, they are seen as promising biomaterials. They exhibit flexibility and softness, which are attributed to their capacity for water absorption [39].

In recent years, hydrogel composites have attracted much attention from the biomedical research community. Natural polymers, including alginate, hyaluronic acid, chitosan, and gelatin, are chosen for their biocompatibility and bioactivity; these fundamental components are integrated into these composites. Because of its adaptability and relative simplicity of modification, gelatin methacrylate (GelMA) has widespread applications [40,41,42,43,44]. Polyethylene glycol (PEG), polyvinyl alcohol (PVA), and poly (lactic-co-glycolic acid) (PLGA) are among the commonly used synthetic polymers [45,46,47].

Because of their adaptability in terms of mechanical characteristics and degradation rates, certain materials are selected for particular biomedical uses. To further enhance hydrogels’ osteoconductive and antibacterial capabilities, inorganic components like hydroxyapatite, bioactive glass, and metallic nanoparticles [44,48,49,50,51] are incorporated. This highlights the significant emphasis on composites developed for bone tissue engineering and infection control.

In general, hydrogels exhibit shear thinning behavior and are not Newtonian fluids. As the applied shear rate increases, the viscosity of materials with shear-thinning behavior decreases in contrast to Newtonian fluids. Injection, medication delivery, and tissue engineering are just a few of the many uses for this crucial property of hydrogels [52].

One typical rheological test for hydrogel characterization is the strain sweep test, also called an amplitude sweep, which involves a steady increase in oscillatory strain. The hydrogel’s storage (G’) and loss (G”) modules over a rising strain range express the results of a strain sweep test, which also provide insights into the material’s Newtonian behavior or linear viscoelastic region (LVR) [53]. At a low shear stress, the hydrogels’ LVR takes place, and the moduli remain constant as stress increases. Additionally, the G’-G” crossover point may approach the gel–sol transition and fluid behavior of the material as stress increases [53].

Among the many rheological methods available, time sweep is one of the most common for tracking the structural evolution of a material through time. One way to assess the hydrogel’s stability is to track changes in its rheological behavior over time. Two kinds of agar hydrogels were subjected to a 60-min oscillatory time sweep test by Bertasa et al. to ascertain their stability. The results show that the hydrogels exhibited high stability, making them relevant for certain uses and highlighting the significance of the time sweep test [54].

A rheological characterization method that sheds light on the hydrogel’s structure when exposed to a specific temperature range is the temperature sweep (temperature ramp) test [55]. Hydrogels’ thermal stability can be tracked with time sweeps. The frequency sweep test is another rheological technique that finds the correlation between testing frequency and storage (G’) and loss (G”) moduli. Furthermore, comparing the two G’ and G” values over the frequency range provides insights into the viscoelastic characteristics and state of a material [56,57].

To determine how well hydrogels work as injectable medication delivery tools, Chen et al. used hydrogels with lower viscosity, loss (G”) and storage (G’) moduli, and frequency and strain sweep and showed that 5% hydrogels flowed more easily and had a lower yield strain than 7.5% hydrogels, suggesting that 5% hydrogels could be a better option for injectability [58].

## 4. Hydrogel Classification

### 4.1. Natural, Synthetic, and Semi-Synthetic

Hydrogels result from the crosslinking of polymer chains. Hydrogels can be categorized based on their sources into three main types: natural, synthetic, and semi-synthetic polymers. In the realm of polymers, hydrogels can be classified as natural, synthetic, or semi-synthetic hydrogels [59].

Cellulose, chitosan, collagen, alginate, agarose, gelatin, hyaluronic acid, and fibrin are all examples of hydrogels that originate from natural sources (natural hydrogels) [60,61]. Their biodegradability, bioactivity, and biocompatibility are all intrinsic properties; however, they lack mechanical strength and stability. Most people can safely use natural hydrogels, but in extremely rare instances, some of the ingredients can cause allergic reactions.

The building blocks of synthetic hydrogels are human-made polymers such as polyvinyl alcohol (PVA), polyethylene glycol (PEG), polyethylene oxide (PEO), poly-2-hydroxyethyl methacrylate (PHEMA), poly-N-isopropyl acrylamide (PNIPAM), polyacrylic acid (PAA), and polyacrylamide (PAAM) [62,63,64]. Their mechanical strength and stability belie the fact that some of them are biocompatible (PAAM, for example). By utilizing closely monitored synthetic procedures, it is possible to create synthetic polymer hydrogels that possess well-defined chemical structures, molecular weights, exceptional mechanical strength, and microstructures that may be customized [65]. Because of their lack of biocompatibility and biodegradability, synthetic polymer hydrogels are not a good choice for cell support [66].

To create semi-synthetic hydrogels, scientists chemically alter natural polymers or use a mix of natural and synthetic polymers. Semi-synthetic hydrogels are a type of hydrogel that is formed by chemically modifying natural polymers or by blending natural polymers with synthetic ones. This combination results in a material that has characteristics of both natural and synthetic hydrogels. By altering natural polymers or mixing them with synthetic ones, scientists create semi-synthetic hydrogels with improved properties, such as enhanced strength, elasticity, or biocompatibility, making them more versatile for specific applications. This type of polymer is known as a semi-synthetic polymer. Chemically modified natural polymers include substances like AcHyA [67] and methacryloyl-modified gelatin (GelMA) [68]. They can also be a mix of natural and synthetic polymers, including gelatin and albumin, or PEG-conjugated fibrinogen [69]. With their varied chemical characteristics, these hydrogels not only mimic the bioactivity of natural hydrogels but also offer a wide range of adjustable qualities [69].

### 4.2. Conventional Hydrogels, Smart Hydrogels, and Nanogels

**Figure 3:**
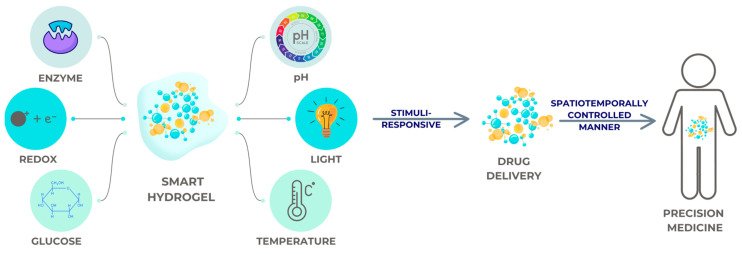
The action mechanisms of a smart hydrogel.

A hydrogel with a focus on biological applications was originally identified in 1960 [70]. Hydrogel systems have come a long way since then. Through this advancement, hydrogels have gone from being “conventional” gels to “smart” gels that can react to specific environmental changes or stimuli, as seen in Figure 3.

Traditional hydrogels are hydrophilic polymers that are lightly crosslinked and swell considerably when exposed to water but remain insoluble. They typically do not have any charges attached to them, and their swelling does not react significantly to changes in the electric field strength, light, temperature, or pH [71,72].

The controlled and sustained release of drugs, together with smart hydrogels’ stimulus-responsive features such as minimal invasiveness, ease of administration, and prominence in drug delivery, has been an important topic in the past several decades [73,74,75].

While conventional hydrogels do not respond to changes in light, electric field, temperature, or pH, smart hydrogels can show large volume changes in reaction to these subtle changes. If they are charged, they have ionic groups that are sensitive to changes in pH, and they typically have a significant hydrophobic component [76,77].

**Figure 4:**
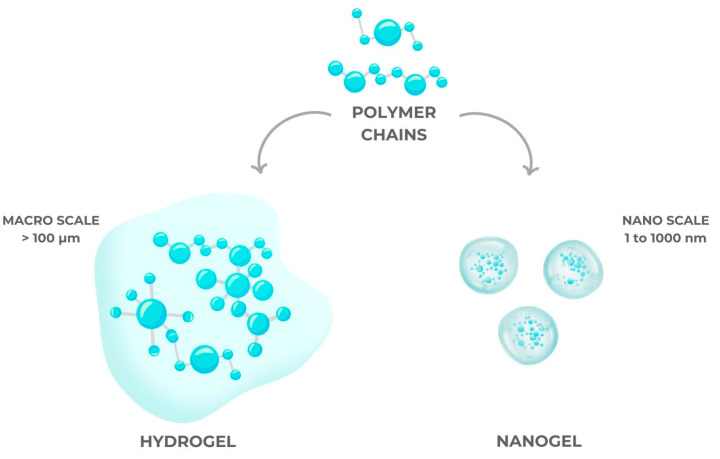
Structures of hydrogels and nanogels.

Because nanotechnology offers a variety of practical solutions to scientific and medical challenges, it is employed in many scientific disciplines. Nanoparticles have been the center of attention in recent years [78]. This demonstrates the great demand for nanotechnologies and knowledge of nanoscale material properties, and the differences between conventional gels and nanogels are illustrated in Figure 4.

When hydrogel components with nanometer-sized fillers are combined, the resulting material is called a nanogel [79]. This type of gel typically has enhanced mechanical qualities or new capabilities. Recent discoveries have highlighted the immense potential of this type of hydrogel for use in numerous biomedical and technical fields thanks to its simplicity in design and synthesis [80].

The unique qualities of nanogels, including their nano-size and hydrogel characteristics, make them highly promising candidates for DDSs [81,82,83], even though hydrogels and nanogels are quite similar. Hydrogels have several desirable characteristics when their particle size is reduced to the nanoscale, including increased mechanical strength, higher reactivity to external stimuli, and exact control over their interfacial properties.

Tissue engineering, cell cultures, and novel drug delivery systems (DDSs) are just a few of the many fields that smart hydrogels are useful in [84]. These hydrogels are perfect for treating diseases because they may release active chemicals in response to physical, chemical, and biological stimuli. Hydrogels are considered ideal for medical applications because their mechanical characteristics, like flexibility and strength, are similar to those of biological tissues such as skin, muscles, and cartilage [85,86]. This similarity allows hydrogels to effectively mimic or simulate the behavior of real-life tissues.

## 5. Innovative Systems for Drug Administration

Hydrogels have a wide range of applications because of their unique structures and how well they work in various environments. Hydrogels’ adaptability, brought about by their water content, opens a world of possibilities in fields as diverse as medicine and dentistry. Their biocompatibility and potentially non-toxic chemical behavior in biological settings further broaden their scope of use in medicine [11].

Controlled drug delivery systems (DDSs) have been utilized to administer pharmaceuticals at certain rates for predetermined durations, overcoming the constraints of standard medication formulations. Hydrogels’ porous architecture makes them very permeable to various medications, allowing for their loading and, under the right circumstances, the release of these substances [12].

Biomaterials exhibiting exceptional performance as drug delivery platforms are in high demand, but an appropriate drug delivery system (DDS) can be a potent therapeutic tool for the complete and efficient treatment of oral illnesses [1]. The most common forms of traditional DDSs include lozenges, pills, and oral gels.

The binding between a loaded medication and the hydrogel matrix can be improved by chemical (covalent bonding) and physical (electrostatic) interactions, allowing for the drug release time to be extended [12]. By using hydrogels, a variety of medications can be protected from potentially harmful conditions, stored, and released at a controlled rate. Physical stimulation from a distance, changes in local temperature or pH, or the presence of certain enzymes can all trigger the on-demand release of drugs [60].

Everyday functioning depends on good oral health, although many people experience some form of oral disease. The need for dental biomaterials is on the rise because of the advancements in oral tissue engineering. When treating oral disorders, it is common to both combat bacterial infections and encourage tissue growth. Hydrogels have enormous promise as drug delivery vehicles and tissue engineering biomaterials to restore damaged oral tissues [60].

Because of their biocompatibility and shape memory properties, hydrogels are a fantastic material for medication administration and tissue regeneration. The internal network of hydrogels creates numerous microscopic gaps and spaces, giving them a typically porous structure [65]. By manipulating the environment and preparation procedure during gel synthesis, one can regulate the size and shape of these pores and voids. Pore structure regulation allows for tissue-specific pore matching, which enhances cell adhesion and proliferation [66].

Furthermore, hydrogels show remarkable antibacterial capabilities when used in the mouth for therapeutic purposes. First, they take physical measures to separate bacteria, which stops the invasion and subsequent spread of dangerous germs. In addition, hydrogels are great drug release carriers, which allows for the efficient distribution of different antibacterial compounds [31,32].

The most important route for therapeutic agent delivery for oral illnesses has traditionally been systemic administration; nevertheless, this approach has drawbacks, including the development of drug resistance, dysbiosis, and adverse effects such as impairment in renal and hepatic functioning [87]. The endodontic tissue that is created using injectable hydrogel scaffolds offers numerous advantages due to the root canal’s tiny size and complex structure. All root canals can be reached by injecting hydrogels. The plasticity and quick absorption of hydrogels into surrounding tissues have gained attention in periodontal and maxillofacial bone tissue engineering. Hydrogels can also mimic the elasticity modulus of soft tissues, allowing them to adhere to the oral mucosa. Therefore, there has been an upsurge in research into hydrogel materials’ potential applications in dentistry among the biomaterials community.

Some potential solutions to these problems include drug carriers, which can (i) control and target drug release, (ii) improve drugs’ pharmacokinetics, and (iii) increase drugs’ bioavailability and selectivity, leading to improved treatment efficacy [88,89,90]. Examples of such carriers include nanoparticles [91,92], hydrogels [93], microparticles [94,95], carbon-based polymers [96], and cyclodextrin-based delivery systems [97]. Drug interactions with different bodily tissues and the safety of delivery can both be enhanced by these carriers [88].

A growing number of dental professionals are considering using nanoscopic drug delivery systems (DDSs) as medication carriers [88]. Mesoporous nanoparticles, nanospheres, nanofibers, core–shell, and nano capsules are just a few structural kinds of nanoparticles [98,99]. Oral infectious pathogen control has made use of a variety of DDSs, including nanoparticles, microparticles, and hydrogels. Most injectable DDSs are hydrogels, which are made using the sol–gel technique [91]. Because of its porous structure, a DDS can pack a considerable amount of medication. Its propensity to swell in an aqueous environment allows for the controlled release of the drug.

It is also possible to tailor these DDSs to react to variations in the oral cavity’s physicochemical parameters, including temperature and pH [91]. A pH-responsive carrier loaded with naringin was produced by Chang et al. [100]. This carrier could react to changes in both temperature and pH. A fluid mixture of carboxymethyl-hexanoyl chitosan, β-glycerol phosphate, and glycerol was used to create the hydrogel, which quickly solidified at 37 °C. Hydrogel naringin release could be accelerated at pH 5.5 because amine groups protonate at an acidic pH [100].

A cellulose-based hydrogel loaded with chlorhexidine was created and tested for drug loading efficiency and hydrogel efficacy in controlling drug release by Tarawneh et al. [101]. With a 58% drug loading efficiency during synthesis, the hydrogel was found to be capable of gradual drug release, with less than 10% of the loaded drug being released after 96 h [101].

### 5.1. Drug Delivery in Periodontal Diseases

**Figure 5:**
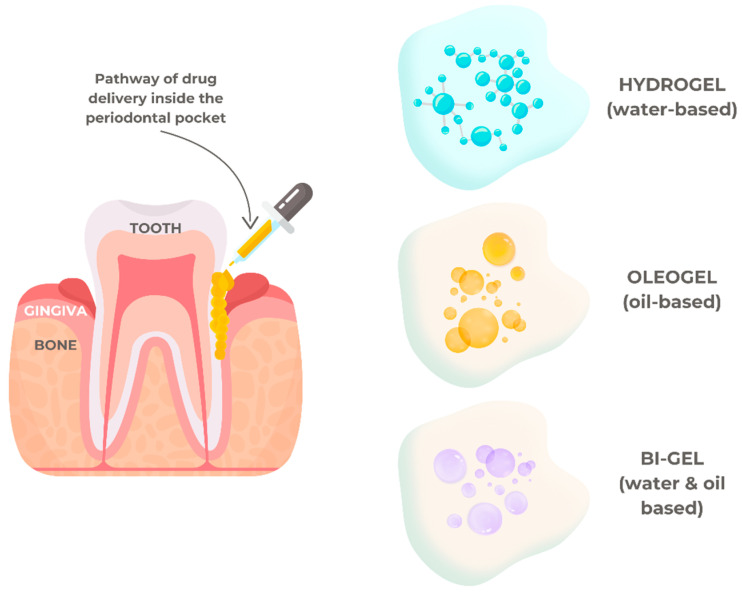
The pathway of a drug delivery system to treat periodontal disease.

Periodontal therapy targets mechanical debridement to eliminate built-up biofilm, disinfect the pocket area, resolve inflammation, and instill ideal hygiene habits in patients. Eliminating or controlling the microbial biofilm and restoring the health and function of the periodontium are the primary objectives of periodontal therapy. A low-viscosity fluid would be ideal for intra-pocket distribution since it would allow for improved penetration at infection sites [102]. Additionally, the system needs to be sticky enough to stay in the periodontal pockets. To address these challenges, various drug delivery systems have been explored, including hydrogels, oleogels, and bigels. Hydrogels are effective for localized drug delivery due to their high water content and biocompatibility, providing a moist environment that promotes healing and sustained drug release. Oleogels, on the other hand, are lipid-based systems that offer a prolonged release of hydrophobic drugs and improved retention in the pocket due to their enhanced adhesiveness. Bigels, a hybrid combination of hydrogels and oleogels, offer a unique pathway for drug delivery by combining the advantageous properties of both systems—hydrophilic and lipophilic phases—allowing for dual-drug encapsulation and release profiles tailored to address the complex microbial environment of periodontal disease. The best mechanism of a drug delivery system to treat periodontal disease is illustrated in Figure 5.

Fibers, stripes, films, and microparticulate systems are among the several delivery methods that can be used to treat periodontitis. Because there are a lot of problems with these systems, the use of gels as local drug delivery systems has garnered much interest for periodontal applications among others [102].

Gels can conform to various periodontal contours, are simply administered, and are biocompatible. When treating periodontitis, hydrogels composed of xanthan gum, polyacrylic acid, sodium carboxymethyl cellulose, chitosan, and polyethene oxide are commonly used to administer non-steroidal anti-inflammatory medicines (NSAIDs) and antibacterial agents [103]. Hydrogels are very biocompatible and mucoadhesive; they stick to periodontal pocket mucosa and are rapidly eliminated by normal catabolic pathways [104]. Rapid drug release, a low mechanical strength, poor stability, and difficulty including hydrophobic medicines are some of the downsides of hydrogels [104,105].

As a possible local drug delivery technology that can sustain high drug levels in the gingival crevicular fluid for extended periods, in situ forming gel (ISG) holds great promise for achieving the desired clinical effects [106].

Gopalakrishna and colleagues recently created an in situ gel filled with piperine [107]. Gellan gum that was deacylated and crosslinked with sodium tripolyphosphate and poloxamer-407 was used to evaluate various gel compositions. The anti-inflammatory efficacy of the improved formulation was assessed after 14 days of implantation in human subjects. An efficient residence period of the hydrogel within the defect was achieved when the hydrogel could develop under physiological circumstances. Additionally, compared to the control group, there were notable decreases in the mean plaque score, gingival index, pocket depth, and anti-inflammatory potential [107].

Gelatin is composed of a combination of peptide sequences. Although it dissolves in hot water, it can still form simple gels at low temperatures through hydrophobic crosslinking. Because of its one-of-a-kind physical and chemical properties, gelatin is both biocompatible and non-immunogenic, making it an ideal transporter for drugs and cells. Water-soluble, non-immunogenic, and gelatin-based hydrogels are promising new classes of medical materials [108,109]. A low-temperature injection anti-inflammatory and antibacterial viscous hydrogel based on punicalagin, hydroxyethyl urea, and gelatin is the subject of the granted patent CN113230448B [110].

As a copolymer, chitosan is made up of a mixture of N-acetyl-Dglucosamine and linear β-1,4-D-glucosamine units that are linked together via β-(1→4) glycosidic bonds [111]. As the sole known naturally occurring cationic polymer, it is a positively charged polyelectrolyte and belongs to the class of polysaccharides that are found in nature. Researchers have investigated chitosan hydrogels for a variety of biological uses, the most common of which being drug delivery systems [112].

As per patent EP3317326B1, chitosan in hydrogels helps provide the necessary rheological characteristics to the gel’s structure [113]. These hydrogels are syringe-injectable because they are thixotropic and biocompatible. Hydrogels, once embedded in the syringe’s body, undergo liquefaction when subjected to shear stress across the plunger; the resulting fluid then flows out of the syringe’s exit port, into the needle, and perhaps into the catheter. It either does not flow and stays put at the application site to release one or more active chemicals (like ciprofloxacin) or it rebounds to form a solid hydrogel for at least a few hours [113].

Swain et al. created an in situ gel containing moxifloxacin hydrochloride to treat periodontitis [114]. A proprietary in situ gel containing doxycycline hyclate was created by Ranch and colleagues using poloxamer 407, chitosan, and polytethylene glycol 600 [115]. The optimal in situ gel’s gelation temperature was 34 ± 1 °C after testing, and it had a good enough textural profile and strength for periodontal applications.

Periodontal abnormalities were treated using hydrogels made of hyaluronic acid and infused with human fibroblast growth factor 2 [116]. Periodontal wound healing clinical indicators increased considerably in 30 individuals after 1 year of treatment [116]. After three months, patients receiving non-surgical periodontal therapy using hyaluronic acid hydrogels as adjuvants had better clinical outcomes according to Olszewska-Czyz et al. [117].

Alternatively, Tamura et al. documented the therapeutic benefits of bFGF sustained release from gelatin hydrogels in patients exhibiting bone abnormalities due to periodontal disease [118]. Clinical indicators, including radiographic bone fill, clinical attachment gain, and a reduction in the probing pocket depth, showed significant improvements one year following therapy. In addition, there were no negative side effects [118,119].

One of the most common types of animal protein found in nature is keratin. Despite keratin’s long history of structural and biological applications, it has received little academic attention due to its challenging extraction procedure. Biomass materials are encompassed under patent CN110511405B [120]. Quick synthesis under moderate circumstances, high yield, easy separation, and purification are all part of the preparation process, which also incorporates the straightforward reaction of grafting alkenyl quaternary ammonium salt. It was ensured that no oligomers were formed throughout the reaction process that was mediated by free radicals. The hydrogel that was made from the obtained grafted keratin is effective against both Gram-positive and Gram-negative bacteria, and it also retains its gel-forming capabilities [120].

Several scaffold forms, such as membranes, sponges, fibers, 3D-printed scaffolds, and hydrogels, have been used to support tissue regeneration and healing in periodontal regenerative therapies. Among the many benefits that hydrogels provide over other scaffold types are tissue mimicking and prolonged medication delivery [107]. Plus, hydrogels can mimic the mouth’s natural moisture balance. Hydrogels provide targeted delivery and localized storage of restorative materials [117]. This keeps the treatment focused on the exact location of the problem and reduces the risk of off-target consequences.

### 5.2. Drug Delivery in Dental Caries

The remineralization of dentin and enamel utilizing bioactive peptides has recently been explored as an alternative to traditional treatment regimens that involve fluoride and amorphous calcium phosphate-based products. Enamel remineralization involves restoring minerals like hydroxyapatite to tooth enamel. This process is crucial for stopping and fixing early stages of tooth decay.

Regulated release and extended contact with the tooth surface made possible by hydrogels make them an ideal delivery vehicle for peptides. An essential component of successful remineralization is the microstructural self-assembly of gels in thin films, which necessitates in-depth research.

A bioactive hydrogel containing the leucine-rich amelogenin peptide (LRAP) was created by Muntean and colleagues [121] with the goal of remineralizing enamel. The capacity of peptides to enhance cell adhesion and treat early carious lesions was highlighted by all investigations. Finally, hydrogels containing short-chain peptides have potential applications in both personal and occupational settings.

A two-pronged strategy for restoring demineralized enamel caries using a mixture of fluoride and a peptide generated from amelogenin was studied in vitro by Ding et al. in [122]. Applying a hydrogel containing chitosan and peptides generated from amelogenin can stimulate enamel healing according to Mukherjee et al. [123]. This hydrogel shows equivalent efficacy to the full-length amelogenin–chitosan hydrogel.

Similar to enamel remineralization, the remineralization and regeneration of dentin have attracted many scholars’ attention, including through the use of hydrogel systems with different characteristics. To design scaffolds that resemble the characteristics of natural mineralized tissue for dentin regeneration, Campodoni et al. [124] used gelatin and chitosan as a hydrogel matrix, adding synthetic mineralized flakes similar to natural mineralized tissues, which were made of gelatin and magnesium-doped hydroxyapatite (MgHA) nanocrystals. Ren et al. [125] developed a hydrogel system by combining amelogenin-derived peptide QP5 with antibacterial chitosan. This system demonstrated an outstanding remineralization effect and good antibacterial capabilities.

Biomimetic remineralization has shown promise in preventing caries through organic and inorganic interactions, but there are currently just a few solutions that meet clinical criteria. Many different approaches, including hydrogel-driven mineralization and protein/peptide-induced mineralization, have been tried thus far to remineralize and restore hard tooth tissue. However, these approaches cannot replace the real hard tooth tissues’ intricate structure entirely.

### 5.3. Drug Delivery in Dental Pulp Regeneration

Endodontic treatment, which involves shaping, cleaning, and filling the pulpal soft tissue space within the tooth with a biocompatible inert material, causes pulp vitality to be lost and compromises the integrity of the tooth structure [126].

One potential strategy in tissue engineering for regenerating dentin–pulp complexes is injecting hydrogels mixed with stem/progenitor cells due to their adaptability to the shape of the pulp chamber and ease of injection inside the tooth. They may effectively fill any irregularity or flaw because their gelation in situ is caused by the crosslinking of hydrogel precursors [127]. At injection time, the material should be able to flow; once in situ, it should harden in reaction to changes in the light, temperature, pH, enzyme, or crosslinking agent [128].

A hydrogel made of polylactic acid and polyethylene glycol (PLGA) was also utilized for the effective apexogenesis of a 20-year-old patient’s lower-left second premolar, which had an immature apical and thin radicular dentinal wall [129]. An injectable hydrogel containing carboxymethyl-chitosan, diglycidyl ether, and calcium phosphate nanoparticles was studied by Osmond et al. [130] for its ability to preserve exposed dental pulp. The findings prove that the hydrogel system is safe for human bodies and helps stimulate the growth and maturation of stem cells from the remaining pulp tissue of teeth.

The regulated release of medicines and an enhancement in the chemotaxis and regeneration potential of human dental pulp stem cells (hDPSCs) were achieved by Soares et al. [131] by incorporating simvastatin into chitosan hydrogels. To stimulate pulp regeneration, the injectable thermosensitive hydrogel PF127 was enhanced by adding the medication iloprost, which can enhance VEFG expression [132].

Research into hydrogels as a means of intracanal drug delivery and their potential uses in pulp capping, root canal disinfection, and pulp regeneration has been extensive due to hydrogels’ highly adaptable properties, particularly those pertaining to injection, which make them well suited to act in the small space within the root canal [133,134].

### 5.4. Drug Delivery in Maxillofacial Diseases

Damage to the maxillofacial bone can range from mild to severe, and it typically manifests itself after teeth extraction, implant placement, and surgery. There has been significant interest in using hydrogels for mandibular reconstruction because of their remarkable biocompatibility, controlled biodegradability, and structural integrity [135]. Injectable hydrogels created by physical or chemical crosslinking are the most popular hydrogel matrices for investigations involving topical medication delivery in the oral and maxillofacial region.

Trimeric molecules, such as collagen, consist of three interconnected alpha-helices [136]. Although its structure was initially uncovered in 1940, the more recent and improved triple-helical structure has been widely acknowledged since 1955 [137] due to years of research. An approach to creating a collagen-based hydrogel that is responsive to changes in temperature and filled with physiologically active polypeptides to mend articular cartilage and bone deformities is safeguarded by patent CN111184917B [138]. Injectability, high biocompatibility, degradability, and non-immunogenicity characterize the produced hydrogel.

An injectable hydrogel that can rapidly deliver microRNA-222 and aspirin (ASP) to local areas was also developed by Lei et al. [139] with the aim of encouraging stem cell differentiation. The injectable hydrogel combined with MSCs (marrow stem cells) and ASP was seen to have promising material for innervated bone tissue engineering.

As the use of dental implants continues to rise, it is imperative that dentists become experts in the necessary procedures and find effective ways to promote bone regeneration. A hyaluronic acid hydrogel containing and releasing bone morphogenetic protein 2 has been extensively used to enhance peri-implant osteogenesis [140].

Certain insects and spiders use silk, scientifically known as silk fibroin, a naturally occurring insoluble fibrous protein, to construct their cocoons and webs [141]. Under normal circumstances (i.e., at room temperature and pH), silk fibroin is stable. Most organic and aqueous solutions do not dissolve it. Although pure silk fibroin has a delicate texture after film formation, its mechanical qualities are on par with, or even better than, those of several high-performance synthetic fibers [141,142]. The injectable silk fibrin porous hydrogel of patent CN109851819B mimics the molding process of natural silk without the need for a crosslinker [143]. This hydrogel has numerous biomedical uses, including filling defects, accommodating the complex shapes of various wounds, and reducing the adverse effects of implants on bodily tissues. Its unique biological features include biodegradability, biocompatibility, non-immunogenicity, stability, and insolubility.

For the synthesis of Sil-GelMA, Xu et al. [144] designed GelMA hydrogels with silibinin, drawing on silibinin’s antioxidant and anti-inflammatory capabilities [145]. The system’s-controlled release of silibinin improved vascularization and had an anti-inflammatory impact. It offers a potential alternative for avoiding alveolar osteitis by enhancing its antimicrobial, hemostatic, and osteogenic functions.

In blood coagulation, protease thrombin enzymes convert the soluble protein fibrinogen into fibrin, an insoluble, non-globular protein that takes the shape of long fibrous chains. Hydrogels derived from fibrin (fibrinogen) have been used in several applications in recent years, including in scaffolds, tissue culture, and improving bone formation and repair [19]. An optimized three-dimensional network of a fibrin–poloxamer polymer composite hydrogel and a method for its synthesis and application as a scaffold for tissue regeneration are included in registered patent KR101991035B1, which is in the global Espacenet database [146] for fibrin hydrogels.

## 6. Future Perspectives

The presence of interconnections, called crosslinks, prevents the dissolution of the hydrogel network through physical and chemical interactions. This class of biomaterials is known as a coherent system. Hydrogels are composed of a three-dimensional polymer network that contains numerous aqueous phases [1,2,3]. Hydrogels have recently gained popularity as a type of targeted drug delivery system, finding applications in various areas of biomedical engineering and medicine. These include tissue engineering for bone and cartilage, biosensors, electronic and soft robotic components, inflammation relief, and wound and cartilage regeneration [4,5,6].

By avoiding drug buildup in non-target tissues, smart hydrogels in drug delivery systems can decrease the dosage frequency while maintaining the required therapeutic concentration in a single dose and minimizing side effects [15,16]. Plus, smart hydrogels are perfect for extended-release systems that contain pharmaceuticals, and they are easy to prepare [17,18].

The success of a drug delivery system relies on the selection of an acceptable framework. More favorable drug efficacy, enhanced permeability, target specificity, and optimized pharmacokinetics are all possible outcomes of using the right biomaterials for therapeutic purposes. Building pH-responsive drug delivery systems using a combination of polymers is the best way to create reasonable, patient-specific medications that interact with the body. The great target specificity of these polymeric systems allows them to have a decreased dosage frequency and limit off-target drug administration, both of which contribute to a reduction in adverse effects.

With their one-of-a-kind characteristics, hydrogels can tackle some problems in dental treatment that other materials struggle with. It can be difficult for traditional oral therapy procedures like rinses or pills to reach specific areas of the mouth that need medication delivery, such as periodontal pockets or ulcerative lesions. To improve therapeutic efficacy while reducing adverse effects, hydrogels can be designed to release active chemicals at specific areas in a regulated and sustained manner. Conventional materials frequently have poor retention in the mouth, making them unable to achieve localized and extended drug release.

Another issue is that the oral mucosa is constantly moving and wet, making it difficult for many materials, such as pastes or lotions, to adhere to the surface. This results in shorter contact times and less effective treatment. Hydrogels can stay in touch with oral tissues for an extended period due to their exceptional mucoadhesive characteristics. This is important for procedures like administering anesthetics, healing oral wounds, or mucositis that necessitate a prolonged exposure time.

Oral health problems, including inflammation and dryness, are frequent, and many traditional materials fail to offer sufficient hydration or healing-promoting environments. Hydrogels can retain moisture due to their high water content, which facilitates the formation of new tissues and the speedier healing of wounds. Items like ointments and solid matrices do not perform this job well.

On the other hand, the delicate oral environment is particularly vulnerable to the irritant, allergic, or poisonous effects that some conventional materials might elicit. There is a smaller chance of adverse responses with hydrogels because they are typically biocompatible and can be made from non-toxic, biocompatible polymers. A crucial factor for usage in the oral cavity is the ability to customize their composition to guarantee safety and efficacy.

In conclusion, hydrogels present distinct benefits including excellent mucoadhesion, moisture retention, mechanical flexibility, and the capability for controlled and localized drug delivery, rendering them essential for specific oral therapies. These attributes enable them to address challenges that other materials cannot, rendering them an essential component in the domain of oral healthcare.

Due to the lack of a standardized assessment of the original literature’s scientific validity in terms of design, methodology, and findings, it is important to note that this review cannot completely avoid bias and inaccuracies. Nevertheless, the use and development of hydrogels for oral medication administration is an interesting area of study.

## 7. Conclusions

Hydrogels are a good choice for topical applications in the oral environment due to their biocompatibility and unique stimulus-responsive properties, which make them ideal for transporting drugs, cells, and other substances to specific locations. This contrasts with other materials like nanoparticles, nanofibers, and thin films.

Hydrogel systems with improved biocompatibility, reduced synthesis costs, and simplified methodologies for achieving excellent sustained local drug delivery efficacy will continue to be a primary focus for researchers in the future. For complicated oral disorders, there are several hydrogel drug delivery systems that use various materials and synthesis methods; nonetheless, the main obstacles preventing their further clinical implementation may be their high costs, complicated synthesis stages, and harmful biodegradation by-products.

Hydrogels’ biocompatibility and the stability of topical drug administration necessitate further research before they can be used in clinical settings. More clinical trials evaluating hydrogels as drug release platforms for various oral disorders are likely to be undertaken in the near future.

## Data Availability

No new data were created or analyzed in this study. Data sharing is not applicable to this article.
